# Rapid and Nondestructive Sex Differentiation of *Aedes aegypti* Using Infrared Spectroscopy and Discriminant Analysis

**DOI:** 10.1021/acsomega.5c07893

**Published:** 2025-11-07

**Authors:** Marfran C. D. Santos, Jorge L. S. Viana, Gigliane J. S. S. Santos, Renata A. Gama, Anne B. F. Câmara, Kássio M. G. Lima

**Affiliations:** † Science and Technology of Sertão Pernambucano, Chemistry Coordination, Rua Projetada, Federal Institute of Education, S/N. Caetano II, 56400-000 Floresta, Pernambuco, Brazil; ‡ Postgraduate Program in Parasitic Biology, 28123 Federal University of Rio Grande do Norte, Avenida Senador Salgado Filho, S/N, Lagoa Nova, 59078-970 Natal, Rio Grande do Norte. Brazil; § Biological Chemistry and Chemometrics Research Group, Institute of Chemistry, Federal University of Rio Grande do Norte, Avenida Senador Salgado Filho, S/N, Lagoa Nova, 59078-970 Natal, Rio Grande do Norte, Brazil

## Abstract

*Aedes aegypti* (*A. aegypti*) mosquitoes are arthropods that transmit a set of viruses of great relevance to public health, such as Dengue, Zika, and Chikungunya, causing recurrent epidemics in tropical and subtropical regions. This fact raises the need for improvements and enhancements in the entomological surveillance of these species, preventing the occurrence of outbreaks. In this work, the potential of near-infrared (NIR) and mid-infrared (MIR) spectroscopy together with variable selection and supervised classification methods for the differentiation of *A. aegypti* mosquitoes according to their sex was analyzed. 66 mosquitoes were analyzed by NIR and 198 by MIR. Chemometric models of successive projection algorithm–linear discriminant analysis (SPA-LDA), genetic algorithm–linear discriminant analysis (GA-LDA), and partial least squares–discriminant analysis (PLS-DA) coupled with interval selection by the successive projections algorithm (iSPA-PLS-DA) were developed to build models capable of differentiating the spectra between males and females. The results demonstrated that sensitivity and specificity values achieved 93.94%, demonstrating that it is possible to perform the differentiation with both NIR and MIR spectroscopies. Based on sensitivity and specificity values, it was possible to note a better performance of the NIR models compared to the MIR models, with the NIR-GA-LDA model being more efficient than the NIR-SPA-LDA model, while NIR-iSPA-PLS-DA was highlighted with the best classification results. Furthermore, a cost and time analysis against gold standard techniques is also performed in this study, demonstrating the advantage of the tools described here. Finally, new studies should be developed so that in the near future it will be possible to count on portable tools based on spectroscopy in the field of entomological surveillance of these species, seeking to identify regions with a high probability of outbreaks in the future.

## Graphical abstract

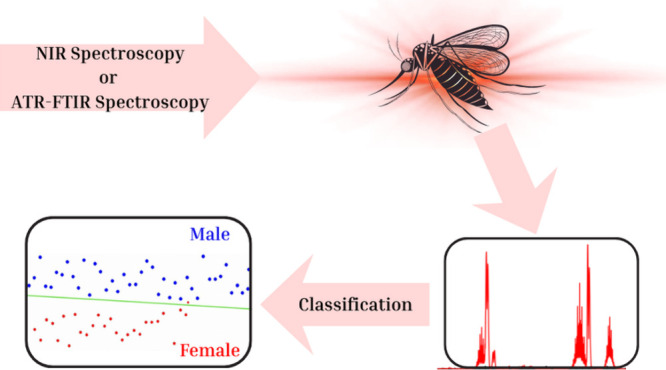

## 1 Introduction

*Aedes aegypti* (*A. aegypti*) mosquitoes are present throughout most of the world and are arthropods that transmit a number of viruses, such as Dengue, Zika, and Chikungunya. These pathogens cause recurring epidemics, especially in tropical and subtropical regions, where climatic and socioeconomic conditions favor the proliferation of the mosquito.^1^ In this context, entomological surveillance emerges as a fundamental pillar for preventing outbreaks, since early detection and elimination of breeding sites can interrupt the mosquito life cycle before viral transmission reaches critical levels.^2^

It is important to note that in the case of *A. aegypti*, only females are hematophagous, requiring blood to develop eggs. This biological characteristic not only sustains mosquito colonies but also makes females the main transmitters of these arboviruses. Therefore, it is important to develop tools that can assist in this monitoring and surveillance process.

Infrared spectroscopy has emerged as a promising solution to these challenges. This analytical technique detects vibrations of chemical bonds in organic molecules, generating spectral “fingerprints” that reflect the biochemical composition of a sample. Both near-infrared (NIR) and mid-infrared (MIR) spectroscopies have been successfully applied to the study of arthropods, including *A. aegypti*, allowing species identification, age determination, and infection status with high accuracy.^3–6^ The advantages of the technique are numerous: it requires minimal sample preparation, provides results in seconds, preserves specimens for later analysis, and can be integrated into portable devices for use in the field.^7^

The spectra obtained by NIR and MIR infrared spectroscopy are a set of multivariate data and therefore can be analyzed using specific techniques for this type of data. Through statistical methods, these techniques can extract relevant information from these data. In this sense, it is possible to use multivariate analysis tools such as successive projection algorithm (SPA), principal component analysis (PCA), genetic algorithm (GA), partial least squares method with discriminant analysis (PLS-DA), linear discriminant analysis (LDA), and quadratic discriminant analysis (QDA), among others. Through them, it is possible to extract important information and discriminate samples according to their spectral information.^2,8–12^

Therefore, this work used near- and mid-infrared spectra in conjunction with variable and interval selection and pattern recognition techniques (SPA-LDA, GA-LDA, and iSPA_PLS-DA) to develop chemometric models capable of differentiating and classifying *A. aegypti* mosquitoes into males and females.

## 2 Materials and Methods

### 2.1 Database

For NIR, the spectra of 66 mosquitoes were collected, 33 females and 33 males, while for MIR, the spectra of 198 mosquitoes were collected, 99 females and 99 males. The mosquitoes were acquired by researchers from the insect and vector laboratory of the Federal University of Rio Grande do Norte (UFRN) in the insectary colonies, where mosquitoes were incubated inside the laboratory from their egg stage to their adult stage. The egg-stage samples were kept at a temperature of approximately 28 °C, a relative humidity of 60%, and a 12 h photoperiod. In the adult stage, the insects were fed a 10% sucrose solution until the time of spectral collection.^2^ The spectra were collected by researchers from the analytical chemistry laboratory of UFRN. As the first collection was carried out in MIR, it was possible to obtain a greater number of spectra compared to the collection in NIR, since, due to the fragility of the sample, some mosquitoes were lost between one collection and another.

### 2.2 Obtaining NIR Spectra

Spectra were obtained using an ARCoptix FT-NIR rocker spectrophotometer (Arcoptix S.A., Switzerland). Readings were taken in transmittance mode with a resolution of 5 nm. A white A4 sheet of paper was used as the surface, and the mosquito was positioned between the sheet and the probe, pressing until a signal was obtained in the abdominal region of the mosquito being analyzed. Spectra were obtained in triplicate by equipment in the 900–2600 nm region. For each new reading, the probe was cleaned with ethyl alcohol (70% (v/v)), a new experimental blank was used, and new paper was used to ensure that there were no traces of the previous analysis. The methodology for acquiring NIR spectra of mosquitoes was developed as described by Santos et al.^2^ The choice of the interval for the analyses was defined by two factors: the first is the biological nature of the sample, where, for the chosen techniques, the determining bands are found in the selected regions; the second point was the agreement with the existing literature in works with similar samples.^2–6^

### 2.3 Obtaining MIR Spectra

Spectra were obtained using a Bruker VERTEX 70 (Bruker Optics, Ltd., U.K.) attenuated total reflection Fourier transform infrared (ATR-FTIR) spectrometer. The spectra were obtained in the range of 600–4000 cm^–1^, with 32 coadded scans and spectral resolution of 4 cm^–1^. The equipment crystal was cleaned with ethyl alcohol (70% (v/v)) for each new collection before the acquisition of the experimental blank and after the sample reading. The analytical blank was performed for air immediately before each spectral acquisition. For the reading of the mosquitoes, aluminum foil surfaces were used between the mosquito and the press to improve data acquisition, pressing it in order to increase the surface in contact with the crystal. The methodology for acquiring MIR spectra of mosquitoes was developed as described by Santos et al.^2^ The choice of the range for the analyses was defined based on the same factors described in Section 2.2, Obtaining NIR Spectra.

### 2.4 Chemometric Methods: SPA-LDA, GA-LDA, and iSPA-PLS-DA

The successive projection algorithm (SPA) is a technique that can be used to select variables in order to eliminate redundant information. In this technique, each variable is considered a vector. Initially, an initial vector (variable) is considered, and new vectors (variables) are implemented with their respective projections in a subspace orthogonal to this initial vector. The SPA criterion for variable selection is the elimination of multicollinearity in the data, that is, the elimination of redundant information. In this sense, the SPA selects those variables that have projections that are more “differentiated” from the others, thus eliminating those vectors that have similar projections.^2,9–13^

The genetic algorithms (GAs) are techniques that can also be used in variable selection. This selection technique imitates Darwin’s theory of evolution. In GA, the initial variables of the population are random; each variable is considered unselected (0) or selected (1). An initial population of variables formed by subsets (chromosomes) of variables is created. In the selection stage, an internal function of the algorithm called the evaluation function (Fitness) assigns a fitness value to each chromosome. Based on this assigned value, the chromosome can be excluded (lowest fitness), duplicated (highest fitness), or kept (average fitness), for example. In the next stage, genetic operators of mutation and crossover are applied to the chromosomes, where crossovers of variables from one chromosome to another can occur, and mutation (a nonselected variable becomes selected or vice versa). This entire process represents a generation. There will be as many generations as requested and, at the end, the selected variables will be chosen within the chromosome that obtained the highest fitness of all chromosomes.^9–12^ For GA, a permutation test was performed in which the classes (males/females) were randomly shuffled several times (permutations). For each permutation, the model was recalculated and its performance was recorded. It was observed that the original model (with the correct labels) performed significantly better than the models with random labels, indicating that the results were not due to chance.

For SPA and GA, the optimal number of variables is determined from the minimum of the cost function *G*, calculated as

1 $$G=\frac{1}{N_{v}}\overset{N_{v}}{\underset{n=1}{\sum }}g_{n}$$

where *g*_*n*_ is given by

2 $$g_{n}=\frac{r^{2}\left(x_{n},m_{I\left(n\right)}\right)}{\min_{I\left(m\right)\neq I\left(n\right)}r^{2}\left(x_{n},m_{I\left(m\right)}\right)}$$

where *I*(*n*) is the true class index for the *n*th validation object *x*_*n*_.

The variables selected by SPA and GA can be used as input information for classification and pattern recognition algorithms. One of the most widely used supervised classification algorithms is the linear discriminant analysis (LDA). LDA computes a function capable of discriminating against classes based on their training data. In the scoring process for classification, LDA considers the approximation that all classes have the same covariance matrix and that the distribution probabilities of all classes are similar.^14^

The successive projections algorithm for interval selection in partial least squares discriminant analysis (iSPA-PLS-DA) is an extension of SPA which aims to select intervals in the spectra. This algorithm comprises two phases, first occurring the division of the spectral responses into intervals of variables, with the creation of different interval combinations, according to a sequence of geometrical projection operations. Sequentially, in phase two, the best combination of intervals is selected on the basis of the error rate yielded by the resulting PLS-DA model.^15^

### 2.5 Software and Model Performance

The computational process of importing, pretreating the data, and constructing the chemometric models was performed using MATLAB R2014b software (MathWorks, Inc., United States). The NIR spectra were preprocessed with a cutoff between 1200 and 2300 nm, EMSC (extended Scarter correction), Baseline (automatic Whittaker filter), and Savitzky–Golay smoothing (15 point window). The MIR spectra were preprocessed with a cutoff between 1800 and 900 cm^–1^, Baseline (automatic Whittaker filter), and Savitzky–Golay smoothing (15 point window). After preprocessing the samples, they were divided into two subsets, one for training (≈70%) and one for testing (≈30%), applying the Kennard–Stone (KS) sampling algorithm.^16^ The models worked with 70% of the samples used in training for later testing with 30% of the samples, that is, external samples that did not participate in training.

The results of the SPA-LDA, GA-LDA and iSPA-PLS-DA multivariate classification models were evaluated based on sensitivity (Sens) and specificity (Spec) calculations. Sensitivity represents the confidence that a female mosquito is identified in a female mosquito spectrum, while specificity represents the confidence that a male mosquito is identified in a male mosquito spectrum. The respective sensitivity and specificity calculations followed eqs 3 and 4:

3 $$\mathrm{Sens}\left(\%\right)=\frac{\mathrm{TF}}{\mathrm{TF}+\mathrm{FF}}\times 100$$

4 $$\mathrm{Spec}\left(\%\right)=\frac{\mathrm{TM}}{\mathrm{TM}+\mathrm{FM}}\times 100$$

where TF = true female; FF = false female; TM = true male; and FM = false male.

## 3 Results and Discussion

The preprocessed average spectra can be seen in Figure 1a for NIR and Figure 1b for MIR. It can be seen that visually the spectra obtained for females and males are very similar, making identification by simple observation significantly difficult, making it necessary to use techniques capable of mathematically analyzing the information for decision making, such as the application of multivariate classification tools. Therefore, the SPA-LDA, GA-LDA and iSPA-PLS-DA models were constructed to classify female and male *A. aegypti* mosquitoes; the results can be seen below.

**Figure 1:**
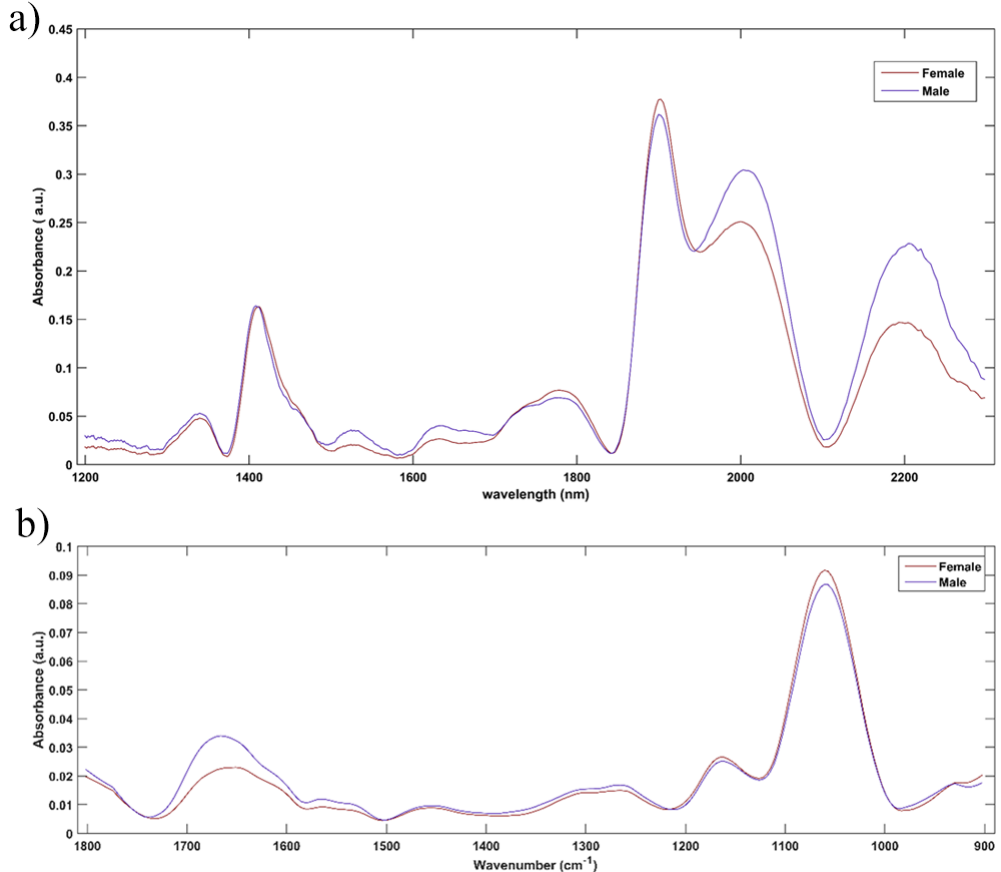
Average spectra obtained in NIR (a) and MIR (b) after preprocessing performed on both spectra. The NIR spectra were preprocessed with a cutoff between 1200 and 2300 nm, EMSC, baseline, and Savitzky–Golay smoothing. The MIR spectra were preprocessed with a cutoff between 1800 and 900 cm^–1^, baseline, and Savitzky–Golay smoothing.

### 3.1 SPA-LDA Model

After training and testing, the SPA selected a total of 5 variables for the NIR and 4 variables for the MIR, as seen in Table 1 and Figure 2.

**Table 1:** Variables Selected by SPA-LDA for the Differentiation of Female and Male *A. aegypti* Mosquitoes

Chemometric model	Selected variables	
NIR-SPA-LDA	1866 nm	1929 nm
	1997 nm	2220 nm
	2298 nm	
MIR-SPA-LDA	1767 cm^–1^	1643 cm^–1^
	1535 cm^–1^	1026 cm^–1^

**Figure 2:**
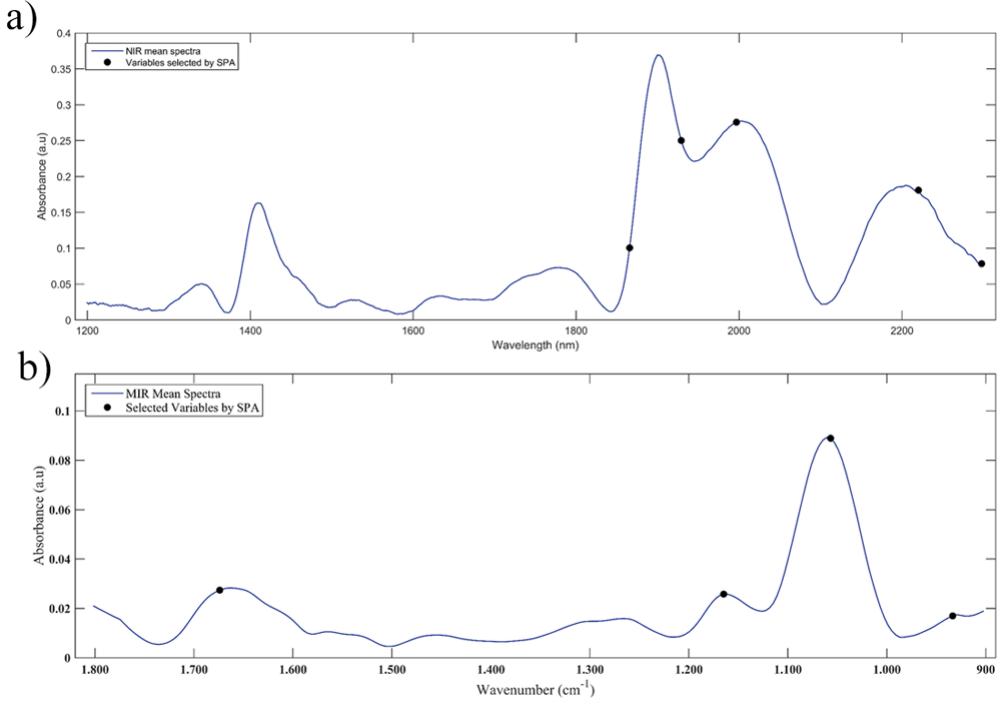
Variables selected by SPA are marked in the average spectrum for NIR (a) and MIR (b).

Among the variables selected by SPA in the NIR spectrum, we can highlight 2220 nm, which is associated with alkenes (CH_2_ stretching and bending), a combination of stretching in N–H C–H bonds; and 2300 nm, associated with methylene (CH stretching and bending).^17,18^ As for the MIR spectrum, the 1640 cm^–1^ region characteristic of amide I and the 1535 cm^–1^ region characteristic of amide II stand out. The presence of these two markings may indicate that the discriminating variation between the classes is the presence of a protein.^17^

Based on the variables selected by SPA, LDA was applied to build a supervised model for mosquito classification. The discriminant function generated can be seen in Figure 3, with Figure 3a being the discriminant function generated for NIR and Figure 3b being the discriminant function generated for MIR.

**Figure 3:**
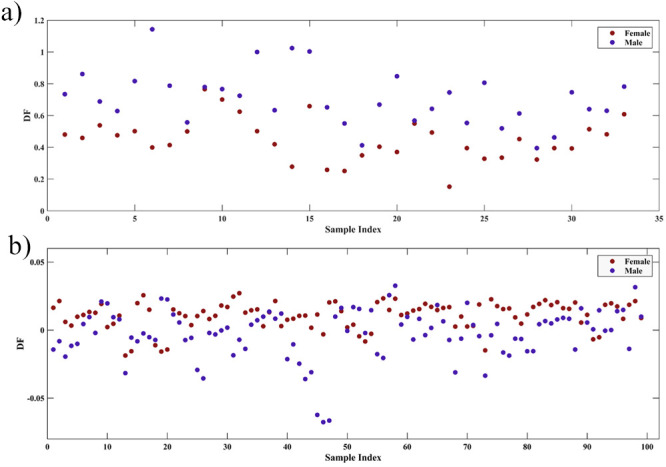
Plot of the discriminant function generated by LDA in the SPA-LDA model for NIR (a) and MIR (b).

Observing the discriminant functions, it is possible to notice a better discrimination of the male and female classes in the NIR-based model when compared to the MIR-based model. After the mosquitoes were classified, sensitivity and specificity calculations were performed for the NIR and MIR models, thus obtaining the values described in Table 2.

**Table 2:** Values Obtained for the Sensitivity and Specificity of the NIR-SPA-LDA and MIR-SPA-LDA Chemometric Models

Chemometric model	Figures of merit
NIR-SPA-LDA	Sens (%): 80.30
	Spec (%): 80.30
MIR-SPA-LDA	Sens (%): 69.89
	Spec (%): 69.89

### 3.2 GA-LDA Model

GA selected only 16 variables for the NIR data, while for MIR 83 variables were selected, which can be seen in Table 3 and Figure 4.

**Table 3:** Variables Selected by GA-LDA for the Differentiation of Female and Male *A. aegypti* Mosquitoes

Chemometric model	Selected variables		
NIR-GA-LDA	1203 nm	1223 nm	1243 nm
	1274 nm	1354 nm	1392 nm
	1474 nm	1478 nm	1478 nm
	1497 nm	1664 nm	1691 nm
	1825 nm	1978 nm	2023 nm
	2126 nm	2189 nm	
MIR-GA-LDA	1794 cm^–1^	1790 cm^–1^	1786 cm^–1^
	1759 cm^–1^	1755 cm^–1^	1751 cm^–1^
	1743 cm^–1^	1736 cm^–1^	1728 cm^–1^
	1724 cm^–1^	1720 cm^–1^	1705 cm^–1^
	1686 cm^–1^	1678 cm^–1^	1670 cm^–1^
	1647 cm^–1^	1643 cm^–1^	1635 cm^–1^
	1608 cm^–1^	1601 cm^–1^	1585 cm^–1^
	1574 cm^–1^	1566 cm^–1^	1562 cm^–1^
	1543 cm^–1^	1535 cm^–1^	1535 cm^–1^
	1535 cm^–1^	1508 cm^–1^	1504 cm^–1^
	1500 cm^–1^	1497 cm^–1^	1481 cm^–1^
	1477 cm^–1^	1473 cm^–1^	1466 cm^–1^
	1435 cm^–1^	1431 cm^–1^	1416 cm^–1^
	1408 cm^–1^	1400 cm^–1^	1396 cm^–1^
	1389 cm^–1^	1385 cm^–1^	1381 cm^–1^
	1369 cm^–1^	1365 cm^–1^	1358 cm^–1^
	1342 cm^–1^	1323 cm^–1^	1311 cm^–1^
	1288 cm^–1^	1284 cm^–1^	1246 cm^–1^
	1238 cm^–1^	1230 cm^–1^	1227 cm^–1^
	1223 cm^–1^	1215 cm^–1^	1203 cm^–1^
	1184 cm^–1^	1180 cm^–1^	1173 cm^–1^
	1157 cm^–1^	1153 cm^–1^	1146 cm^–1^
	1130 cm^–1^	1119 cm^–1^	1111 cm^–1^
	1099 cm^–1^	1088 cm^–1^	1084 cm^–1^
	1068 cm^–1^	1061 cm^–1^	1026 cm^–1^
	1018 cm^–1^	1003 cm^–1^	991 cm^–1^
	960 cm^–1^	953 cm^–1^	937 cm^–1^
	918 cm^–1^	899 cm^–1^	

**Figure 4:**
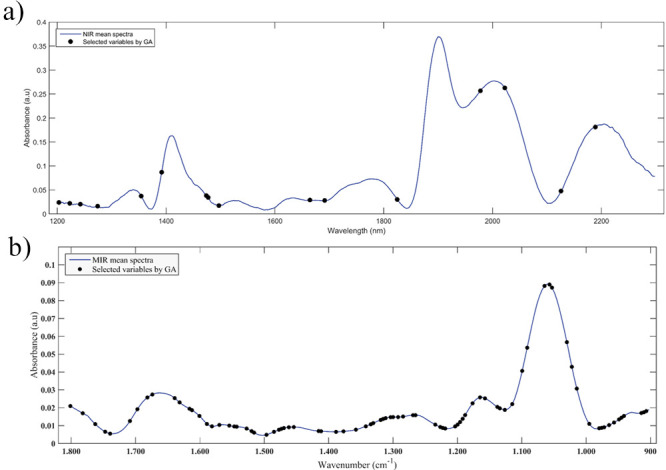
Graphs of the variables selected by SPA-LDA marked in the average spectrum of NIR (a) and MIR (b) of all classes. The variables were selected by GA to discriminate between female and male *A. aegypti* mosquitoes.

Among the variables selected by GA in the NIR spectrum, the following stand out: 1223 nm, which is associated with the second C–H stretching overtone; 1478 nm, associated with the first OH stretching overtone; and 1691 nm, associated with the first C–H stretching overtone in CH_3_ groups. In the variables selected in the MIR spectrum, the wavelengths in the regions 1670 cm^–1^, which are related to the amide I group, and the 1562 cm^–1^ region, which are related to the amide II group, are both repeated when compared to the pattern presented in the SPA model, further reinforcing the possibility that the differentiation between the sexes of mosquitoes is related to some protein.^17^

Then, LDA was applied to generate the supervised model based on the variables selected by GA (Figure 5), and sensitivity and specificity calculations were performed for the models, obtaining the values described in Table 4.

**Figure 5:**
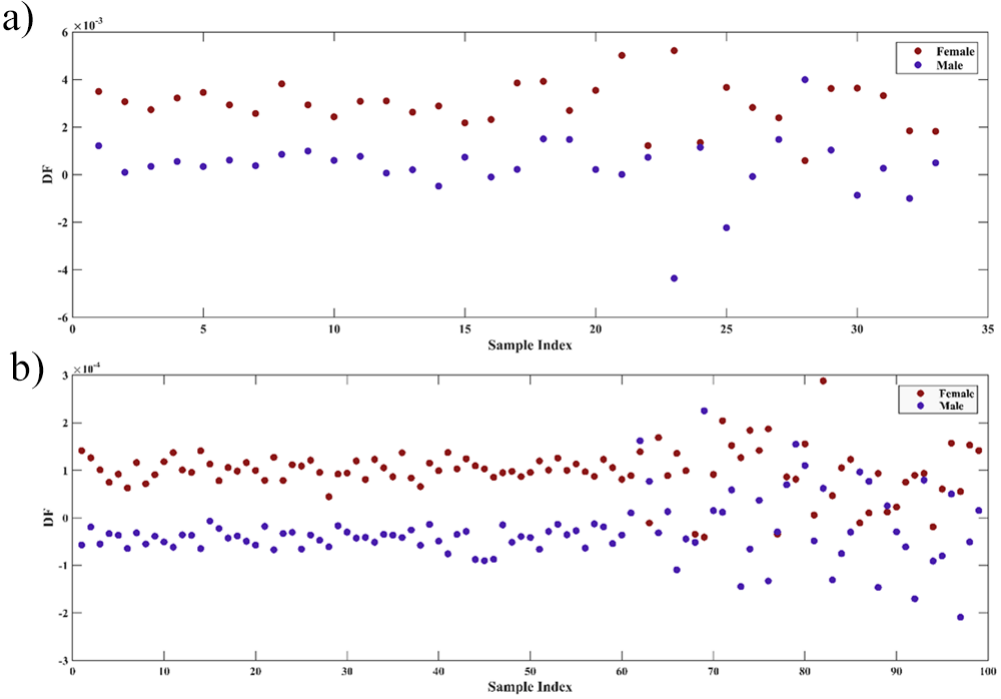
Plot of the discriminant function generated by LDA in the GA-LDA model for NIR (a) and MIR (b).

**Table 4:** Variables Selected by GA-LDA for the Differentiation of Female and Male *A. aegypti* Mosquitoes

Chemometric model	Figures of merit
NIR-GA-LDA	Sens (%): 93.94
	Spec (%): 93.94
MIR-GA-LDA	Sens (%): 88.26
	Spec (%): 88.26

### 3.3 iSPA-PLS-DA Model

Figure 6 depicts the selected intervals for NIR and MIR spectra. iSPA-PLS-DA selected only one interval for NIR spectra, with the wavelength in the range of 1917–1944 nm, which can be associated with the second overtone of amide and the COOH stretching of carboxylic acids.^18^ As for the MIR spectrum, it is possible to highlight the wavenumbers in the range of 1527–1569 cm^–1^, which is characteristic of amide II.^19^ Furthermore, the selection of these intervals for the segregation between male and female mosquitoes probably occurs due to the presence of proteins, in agreement with the variables previously selected in GA and SPA algorithms.

**Figure 6:**
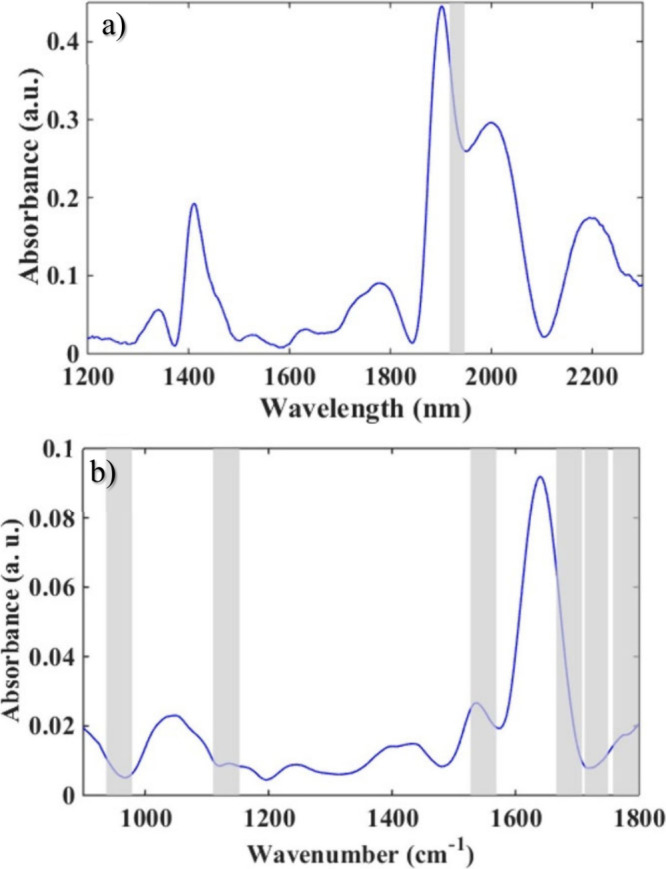
Plots of selected intervals in the iSPA-PLS-DA NIR (a) and MIR (b) models.

Table 5 shows the sensitivity and specificity values calculated for NIR-iSPA-PLS-DA and MIR-iSPA-PLS-DA.

**Table 5:** Values Obtained for the Sensitivity and Specificity of the NIR-iSPA-PLSA-DA and MIR-iSPA-PLS-DA Chemometric Models

Chemometric model	Figures of merit
NIR-iSPA-PLS-DA	Sens (%): 100.00
	Spec (%): 90.91
MIR-iSPA-PLS-DA	Sens (%): 55.17
	Spec (%): 88.10

### 3.4 2D Correlation Analysis

2D correlation spectroscopy was applied in this study to investigate the relationship between different NIR and MIR wavenumbers/wavelengths for the segregation of female and male *A. aegypti* mosquitoes, as depicted in Figure 7. This correlation aims to identify the molecular origin of NIR markers, since MIR bands are sharper and well-documented in specialized literature, facilitating the search for spectral biomarkers.^20^ A correlation marker (highlighted with the black arrow) for both genders of mosquitoes was found at ∼1670 cm^–1^, combined with the ∼1360 nm NIR band, which can be attributed to the amide I group. Another strong correlation could be found at ∼1411 cm^–1^, attributed to stretching of C–N, with the NIR band at 1335 nm.

**Figure 7:**
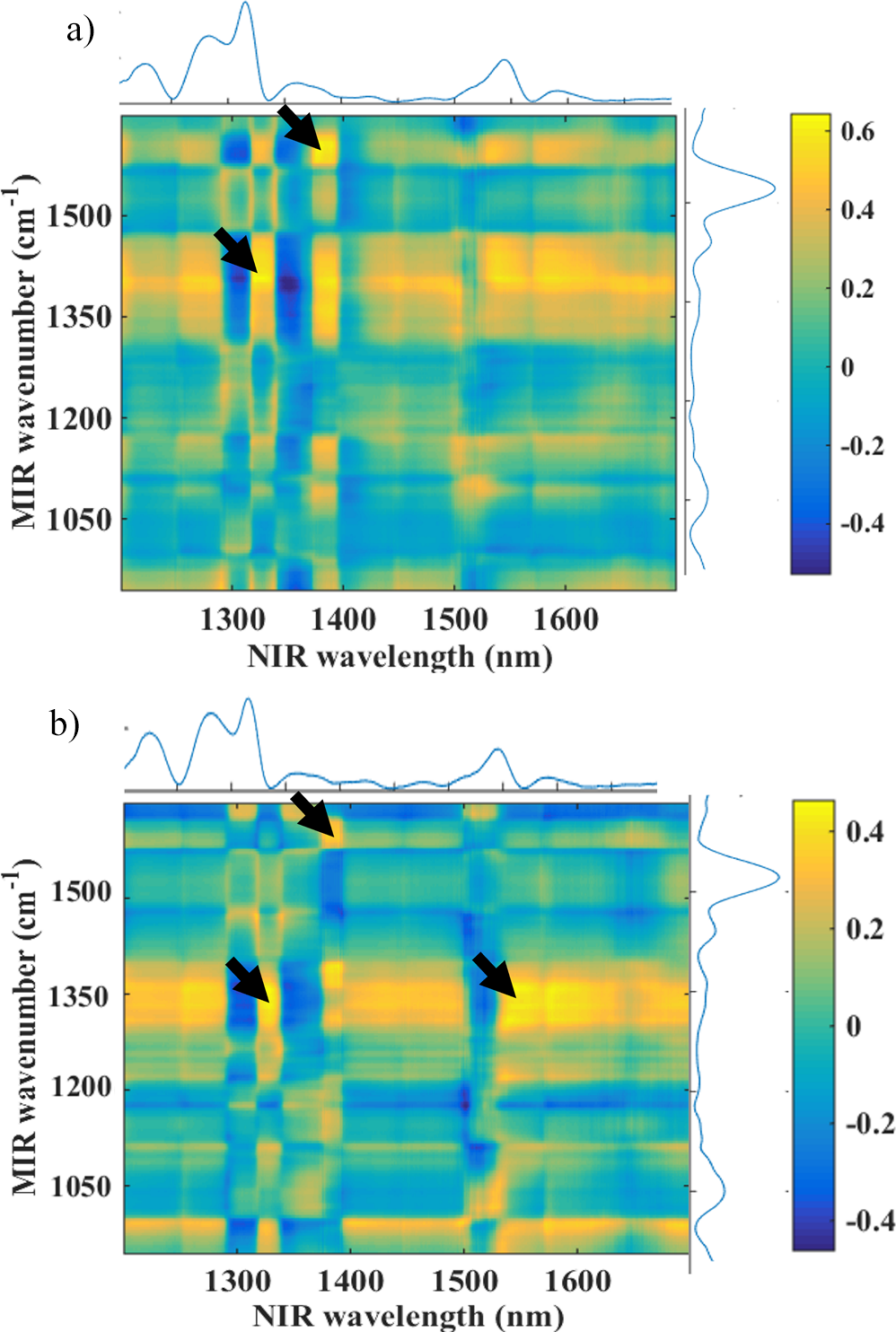
Synchronous map obtained from 2D correlation for female (a) and male (b) *A. aegypti* mosquitoes. Black arrows indicate the correlation between the MIR and NIR spectroscopic bands.

### 3.5 Cost and Time Analysis

In this study, spectral acquisition took about 15 s per sample. In this sense, the time to acquire the spectrum of 100 samples would take about 50 min. For comparison purposes, the process of preparing the mosquitoes, extracting DNA, and performing RT-qPCR for a similar analysis, without considering the time to acquire the reagents, would require 900 min, which would be about 2 full days of work.^21^

Regarding costs, disregarding the acquisition price of the instruments needed to perform each technique (comparing NIR, MIR, and RT-PCR, spectroscopic techniques still have an advantage in cost), the costs for acquiring 100 sample spectra in NIR would be around US$10, since it is a technique that does not require reagents to be performed, its cost would be reduced only to the payment of an analyst’s working hour. In RT-qPCR, the costs of reagents alone for acquiring 1 sample would already be around US$10, considering the acquisition of 100 samples, but the payment of US$10 per hour worked by the analyst would put the total price at approximately $1160.^21^ In these parameters, NIR ends up being 18 times faster and 116 times cheaper than RT-qPCR, thus being a viable option for improving entomological surveillance.

### 3.6 Discussing the Results

The need for identificationof the gender of *A. aegypti* mosquitoes requires complementary tools, since only female’s species are hematophagous requiring blood to develop eggs, which can transmit several viruses, such as, Dengue, Zika, and Chikungunya. In this context, it is important to develop tools that can assist monitoring and the surveillance process of the mosquitoes, since early detection and elimination of breeding sites can interrupt the mosquito life cycle before viral transmission reaches critical levels.^2^ In this study, infrared spectroscopy (NIRS and ATR-FTIR) was applied combined with SPA-LDA, GA-LDA, and iSPA-PLS-DA for the rapid segregation between females and male mosquitoes. The importance of these results lies in the possibility of application portable traps equipped with an NIR spectrophotometer combined with chemometric tools for the prediction of the reproductive growth of colonies, with the aim of preventing these arboviruses.

In this study, NIR and ATR-FTIR were successfully applied in discriminating against the gender of *A. aegypti* mosquitoes. The variables selected by SPA for the NIR spectra (Table 1) indicate that some biochemical changes between the classes can be found at 2220 nm, which is associated with alkenes (CH_2_ stretching and bending), a combination of stretching in N–H C–H bonds, and at 2300 nm, associated with methylene (CH stretching and bending).^17,18^ In relation to MIR spectrum, SPA selected wavenumbers in the region of 1640 and 1535 cm^–1^ are characteristics of amide I and amide II, respectively, while the band at 1767 cm^–1^ is related with lipid structures.^19^ These spectral biomarkers may indicate that the discrimination between the classes is due to the presence of some proteins in the mosquito. In addition, NIR-SPA-LDA achieves a better segregation of scores for each class than MIR-SPA-LDA, with sensitivity and specificity of 80.30%, as observed in Table 2, demonstrating that this model was more successful in the classification.

In relation to the variables selected by GA, it is possible to highlight the following wavelength for NIR: the second C–H stretching overtone in 1223 nm; the first OH stretching overtone in 1478 nm; the band in 1691 nm associated with first C–H stretching overtone in CH_3_ groups. On the other hand, GA selected more specific variables for MIR spectroscopy, such as the wavenumbers related to the amide I and amide II groups, in the wavenumbers of 1670 and 1562 cm^–1^, respectively. The presence of these groups reinforces the possibility of the segregation of mosquitoes being related with some protein. Although the MIR spectra present more specific biomarkers for the differentiation between classes, the best classification results with GA were obtained for NIR-GA-LDA, with specificity and sensitivity of 93.94%, while MIR-GA-LDA achieved a sensitivity of 88.26%.

Furthermore, the infrared data were analyzed by iSPA-PLS-DA. The NIR-iSPA-PLS-DA model was obtained with 4 latent variables and selecting only 1 interval of the spectra, which can be associated with the second overtone of amide and the COOH stretching of carboxylic acids. In MIR-iSPA-PLS-DA, the model was obtained with 2 latent variables and selected 6 spectral intervals, wherein the wavenumber in the range of 1527–1569 cm^–1^ stands out, characteristic of amide II. In this case, iSPA selected variables related to proteins in both IR regions, corroborating with the association of these molecules to the segregation of the classes, in agreement with the variables previously selected in GA and SPA algorithms. The better segregation results for NIR-iSPA-PLS-DA are probably due to the capacity of the iSPA to eliminate the redundant information and chose only that region whose analytical information is useful for the intended purpose, in this case, variables related with proteins.^22^

The application of 2D correlation spectroscopy allowed the study of relationships between different MIR and NIR variables. Markers found in the synchronous map were related to the Amide I group and the stretching of C–N, which matched the variables selected by the chemometric models, demonstrating that NIR bands are correlated with specific ATR-FTIR bands, enhancing the interpretability of NIR spectrum.

In addition, as can be seen by analyzing the results, the models can be ordered in increasing order of potential for use in mosquito sexing as MIR-iSPA-PLS-DA < MIR-SPA-LDA < NIR-SPA-LDA < MIR-GA-LDA < NIR-GA-LDA < NIR-iSPA-PLS-DA. Therefore, we can conclude that, within the same chemometric model, the NIR technique is superior to MIR in the spectral investigation of mosquitoes. Furthermore, when comparing the different chemometric techniques, GA-LDA proved to be superior to SPA-LDA in the classification work, while the best classification model for female and male mosquitoes was iSPA-PLS-DA.

One explanation for the NIR technique demonstrating better results than MIR may be the fact that the spectral acquisition in NIR is performed by a probe relatively compatible with the size of the mosquito, making it possible to couple the mosquito to the probe with the most similar position among all mosquitoes, thus, this analysis reduces random errors. On the other hand, the ATR crystal in MIR spectral acquisition makes the analyst’s work more difficult, and it is likely that small variations will occur in the positions of the mosquitoes, variations that can be translated into the spectra.

It is expected that among the selected variables, variables related to the biomolecules that determine the difference in the sex of the species will be found. In this sense, we believe that one path for this investigation involves the NIX gene.

A recent study revealed the existence of the Nix gene, responsible for initiating male development in the *A. aegypti* mosquito. This gene acts as a primary sex-determining factor, playing a crucial role in the sexual differentiation of the species. The Nix cDNA consists of a sequence of 985 base pairs, which encodes a polypeptide of 288 amino acids. Genomic analyses indicate that the Nix gene is present exclusively in the DNA of male individuals, being absent in the female genome. Its expression occurs at an early stage of development, even before sex determination, suggesting that it plays a fundamental role in activating the male differentiation pathway. Furthermore, Nix is located within the M locus, a specific region of the male sex chromosome of *A. aegypti*.^23,24^

In addition to Nix, two other genes have been identified as essential components of the sex determination pathway in *A. aegypti*: fruitless (fru) and doublesex (dsx). These genes are well-known in several organisms and play regulatory roles in sexual differentiation. In the presence of Nix, the dsx and fru genes undergo an alternative splicing process, resulting in the production of male-specific isoforms. This mechanism allows the activation of genetic programs that direct male development. In the absence of Nix, alternative transcription of the female versions of the dsx and fru genes occurs, promoting differentiation to the female sex.^25^ More in-depth studies must be developed in order to relate the variables selected here with the NIX gene or any other sex-determining biomolecule.

For *A. aegypti* mosquitoes, the main sexual differences associated with significant biochemical variations between males and females are likely related to protein content and composition (related to the greater development of the reproductive system in females); differences in the proportion of lipids and glycogen (related to energy metabolism); structural variations in the cuticle, especially in chitin content; and the Nix, fru, and dsx genes. These components exhibit characteristic absorption bands in the infrared, especially in the C–H, O–H, and N–H stretching regions, as well as in amide I and II vibrations, which are sensitive to protein content. Thus, although there is no direct identification of a single compound at low concentrations, the overall pattern of bands and their relative intensities reflect the differentiated biochemical composition between the sexes. Therefore, chemometric algorithms exploit these multivariate differences across the full spectrum, allowing classification with good accuracy, and we believe these are the variations responsible for the discrimination achieved in this study.

Another point of investigation in this study was the analysis of time and costs. For a response time analysis, the spectral acquisition took approximately 15 s. Therefore, it took approximately 50 min to acquire the spectrum of 100 samples, while it would take 2 days to prepare the mosquitoes, extracting DNA and performing RT-qPCR for a similar analysis. This comparison shows that the methodology based on NIR spectroscopy is about 18 times faster than TR-qPCR. In relation to costs, for acquiring 100 sample spectra in NIR would be around US$10, since it is a technique that does not require reagents to be performed, its cost would be reduced only to the payment of an analyst’s working hour.^21^ In these parameters, NIR ends up being 18 times faster and 116 times cheaper than RT-qPCR, thus making it a viable option for improving entomological surveillance.

Finally, this approach demonstrates the high efficiency of IR spectroscopy in classification of *A. aegypti* mosquitoes, with the advantage of NIR instruments being usually portable, which represents an interesting feature for in situ analysis or the production of traps for prediction of the reproductive growth of colonies.

## 4 Conclusions

This study is one of the first to explore the use of infrared spectroscopy for rapid sex determination of *A. aegypti* mosquitoes, aiming to improve colony management and facilitate their differentiation in research. It also proposes the application of this technique in portable traps equipped with an NIR spectrophotometer, allowing, in conjunction with statistical analyses, prediction of the reproductive growth of colonies. This approach can contribute to the prevention of epidemics and the identification of areas with a greater risk and proliferation of mosquitoes.

Considering this possibility becomes feasible when we consider some factors, such as the portability of the NIR equipment, which would facilitate mosquito analysis, and the time and cost of using the technique compared to other techniques considered the gold standard.

It is worth noting that this study was conducted under laboratory conditions, and further tests are necessary to better validate the technique as well as the use of new classification techniques in order to obtain even higher sensitivity and specificity values. However, we believe that this is the beginning of the application of infrared spectroscopy for colony monitoring and rapid sexing of *A. aegypti* mosquitoes.
